# Ontogeny of sex differences in the energetics and kinematics of terrestrial locomotion in leghorn chickens (*Gallus gallus domesticus*)

**DOI:** 10.1038/srep24292

**Published:** 2016-04-12

**Authors:** K. A. Rose, K. T. Bates, R. L. Nudds, J. R. Codd

**Affiliations:** 1 Faculty of Life Sciences, University of Manchester, Manchester, M139PT, UK.

## Abstract

Sex differences in locomotor performance may precede the onset of sexual maturity and/or arise concomitantly with secondary sex characteristics. Here, we present the first study to quantify the terrestrial locomotor morphology, energetics and kinematics in a species, either side of sexual maturation. In domestic leghorn chickens (*Gallus gallus domesticus*) sexual maturation brings about permanent female gravidity and increased male hind limb muscle mass. We found that the sexes of a juvenile cohort of leghorns shared similar maximum sustainable speeds, while in a sexually mature cohort maximum sustainable speeds were greater by 67% (males) and 34% (females). Furthermore, relative to that in juveniles of the same sex, the absolute duration of leg swing was longer in mature males and shorter in mature females. Consequently, the proportion of a stride that each limb was in contact with the ground (duty factor) was higher in sexually mature females compared to males. Modulation of the duty factor with the development of secondary sex characteristics may act to minimize mechanical work in males; and minimise mechanical power and/or peak force in females. A greater incremental response of mass-specific metabolic power to speed in males compared to females was common to both age cohorts and, therefore, likely results from physiological sexual dimorphisms that precede sexual maturation.

Artificial selection in the domestic chicken (*Gallus gallus domesticus*) has led to derived morphology, physiology and behaviour distinct from that of its red jungle fowl ancestor^1^. Unintended pathological consequences often result from artificial selection in farm animals^2^. For example, in the broiler chicken, selection for increased muscle growth rates has led to a compromise in the effectiveness of the respiratory apparatus^3^, abnormalities of the musculoskeletal system^4^, and the negative ontogenetic allometry of the heart and lungs associated with a number of pathologies^3^. The influence of this type of selection in broilers upon locomotor mechanics^5^,^6^,^7^,^8^ and morphology^9^,^10^, across ontogeny^3^,^11^, and also in comparison to less derived/wild-type strains^9^ is well studied. In layer chickens, however, selected for increased reproductive output (size and frequency of eggs laid), potential changes in locomotor physiology and mechanics associated with the developmental process have not yet been investigated.

The energy budgets of animals are limited^12^, and consequently, trade-offs in resource allocation exist at different life stages^13^. In young animals, a bias in energy is allocated towards somatic tissue growth. An inherent trade-off exists, however, between growth rate and the maturation of tissues required for locomotion^14^,^15^,^16^. Linked to this compromise, avian species exhibit distinct differences in ontogenetic strategy. Precocial birds (usually cursorial), for example, prioritise effective locomotion^17^,^18^,^19^ over growth from hatch and will grow 3–4 times slower than altricial birds (often principally flyers) that do not begin locomotion until after the whole body growth period^14^,^15^,^20^. Other bird groups, with two or more principle modes of locomotion, such as the mallard (*Anas platyrhynchos*), which flies, walks and swims, exhibit differential ontogenetic strategies between the hind- and forelimbs, depending on which mode they use principally in early life^21^. The precocial strategy is thought to have arisen due to the strong evolutionary pressure posed by high predation rates on vulnerable juveniles^22^,^23^, which are handicapped due to small body size, rapidly growing (softer) tissues and naiveté about their environment^24^. Sexual maturation usually occurs later in the ontogenetic trajectory^25^,^26^, at which point, energy no longer required for growth can be invested in reproduction^13^.

Domestic white leghorn (layer) chickens are precocial; however, their energy allocation differs from that of wild precocial birds due to food not being a limiting resource. The ability of the digestive and transport systems of the bird becomes the limiting factor in terms of energy intake and allocation to tissues. These environmental conditions combined with artificial selection have allowed for a shift in energy allocation in the sexes. In females, the emphasis is on reproduction, bringing forward the onset of egg-laying^27^, which is continuous throughout the lives of hens, rather than occurring only in breeding seasons. Male birds on the other hand, invest substantial energy in skeletal muscle tissue allocation, possessing greater muscle, bone, heart and blood masses compared to mature females, which outweigh males in digestive components, skin and fat as well as the reproductive system^28^. These birds, therefore, exhibit distinct sexual dimorphisms in skeletal muscle and reproductive tissue allocation. The sexes, however, share similar initial post-hatch growth trajectories in body mass (*M*_b_)^28^. At the onset of sexual maturity (roughly 4–5 months old), the differentiation of secondary sexual characteristics is mediated by a rise in gonadal hormones and male skeletal muscle growth rates increase relative to females. Female growth also terminates before that of males, leading to strong male-biased sexual size dimorphism^28^.

Sexually mature leghorns exhibit sex differences in energy metabolism during locomotion, whereby the incremental increase in mass-specific metabolic power (*P*_met_, W kg^−1^) with speed (*U*) is steeper in males than in females^29^. It is therefore more metabolically costly for males to move at faster speeds compared to females. Comparison of the energy metabolism of these birds at dynamically similar speeds did not account for the sex difference^29^. The dimorphism in energy metabolism was, therefore, hypothesised to be the result of additional sexual dimorphisms in morphology and/or physiology^29^. Furthermore, males were able to sustain maximum speeds (*U*_max_) approximately 25% greater than those of females^30^. It is unclear whether these differences are associated with female or male specialisations or constraints, or a combination of these. It is also unclear whether these sex differences in locomotion are already manifested in the juvenile form or develop at the onset of sexual maturity. Furthermore, it is unclear whether the onset of continuous gravidity impedes upon the locomotor abilities of hens.

The aim of this study was to investigate the effects of the ontogenetic differences in male and female morphology on the energetics and kinematics of locomotion in white leghorns. To achieve this, two age cohorts were selected for comparison: one prior to the onset of sexual maturity (juvenile [14–16 week-old] males and females: J_♂_ and J_♀_) and another that was sexually mature (≥20-week-old: M_♂_ and M_♀_). Sexual differentiation at maturity is a gradual process and may have already initiated in the younger cohort; however we were able to confirm that the mature cohort displayed male crowing and secondary sexual characteristics (large red combs and wattles) and female egg laying, whilst the juvenile cohort did not. We also quantified the accompanying sex-specific musculoskeletal and reproductive volumes and dimensions. The hypothesis tested was that none of the locomotor differences would be present in the juvenile cohort i.e., the sexual dimorphism in locomotor performance would develop concomitantly with the secondary sex characteristics in these birds.

## Results

### Body mass

*M*_b_ (Table 1) was significantly greater in the sexually mature, compared to the juvenile cohort (Table 2). A significant age × sex interaction in *M*_b_ was identified due to similar masses in the sexes of the juvenile cohort (1.05-fold greater in J_♂_ than in J_♀_), but a 1.34-fold greater *M*_b_ in M_♂_ than in M_♀_ (Table 2). This was associated with a greater difference in *M*_b_ between J_♂_ and M_♂_ (0.82 kg) than between J_♀_ and M_♀_ (0.41 kg).

### Limb bone lengths

All absolute hind limb skeletal bone lengths (Table 1) were significantly longer in males compared to females (Table 2). A significant age × sex interaction was present in the sum of the three hind limb bone lengths (Σ*l*_segs_) due to a greater sex difference in the mature compared to the juvenile cohort. This was linked to a lack of difference Σ*l*_segs_ between J_♀_ and M_♀_; but significantly longer Σ*l*_segs_ in M_♂_ compared to J_♂_ (Table 2).

### Reproductive mass

Reproductive mass in M_♀_ was on average 162.62 ± 25.20 (s.d) g, which comprised 11.49 ± 1.26 (s.d)% of *M*_b_.

### Muscle measurements

**Figure 1:**
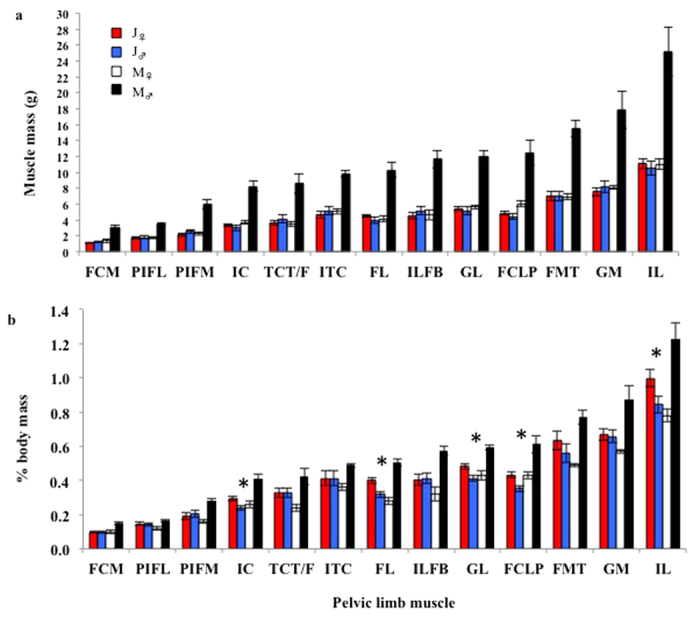
Mean pelvic limb muscle measurements. **(A)** Absolute muscle mass. **(B)** Muscle percentage of total body mass. Muscle abbreviations are defined in Table 3. A significant age × sex interaction was identified in all measurements (Table 2). Asterisks denote where the sex differences are the opposite between the two age cohorts. Error bars represent s.e.m.

In each of thirteen measured pelvic limb muscles (see Table 3 for abbreviations) a significant age × sex interaction was present in absolute mass (Fig. 1A, Table 2), which did not differ significantly between the sexes of the juvenile cohort, but was greater in M_♂_ than in M_♀_ (Table 2). Each muscle was also of similar absolute mass in J_♀_ and M_♀_, with the exception of the FCLP, which was greater in M_♀_ than in J_♀_. Furthermore, each absolute muscle mass was greater in M_♂_ than in J_♂_.

A significant age × sex interaction was also present in the relative mass (%*M*_b_) of each pelvic limb muscles (Fig. 1B, Table 2). In the mature cohort, the relative mass of each muscle was greater in M_♂_ than in M_♀_. In the juvenile cohort, however, the majority of muscles were similar in relative mass between the sexes, with the exceptions of the IC, FL, GL, FCLP and FMT, in which it was greater in J_♀_ than in J_♂_ (the opposite sexual dimorphism to the mature cohort). Since the females of the two age cohorts did not differ significantly in the absolute masses of their individual muscles, the lower %*M*_b_ of the muscles in M_♀_ relative to J_♀_ was due to the greater the *M*_b_ of the M_♀_ relative to J_♀_ (attributed to increased mass in the body outside of the pelvic limb) and linked to gravidity.

Therefore, M_♂_ had greater relative muscle masses than J_♂_ associated with the increase in absolute muscle mass that occurs with male sexual maturation. In opposition, M_♀_ had lower relative muscle masses than J_♀_, associated with the lack of change in muscle mass but increase in reproductive mass that occurs with female sexual maturation.

### Maximum sustainable speed

Juvenile leghorns of both sexes reached a *U*_max_ of 0.83 m s^−1^. In comparison, the *U*_max_ of M_♂_ (1.39 m s^−1^) exceeded that of M_♀_ (1.11 m s^−1^) by 25%. Mature leghorns of each sex achieved greater *U*_max_ than the juveniles: the *U*_max_ of M_♂_ exceeded that of J_♂,_ by 67% and the *U*_max_ of M_♀_ exceeded that of J_♀_ by 34%.

### Standing metabolic rates

Mass-specific *P*_met_ during quiet standing was similar in the males and females within each cohort but was greater in the juvenile compared to sexually mature cohort by ~2 W kg^−1^ (Table 4).

### Metabolic rates during locomotion

**Figure 2:**
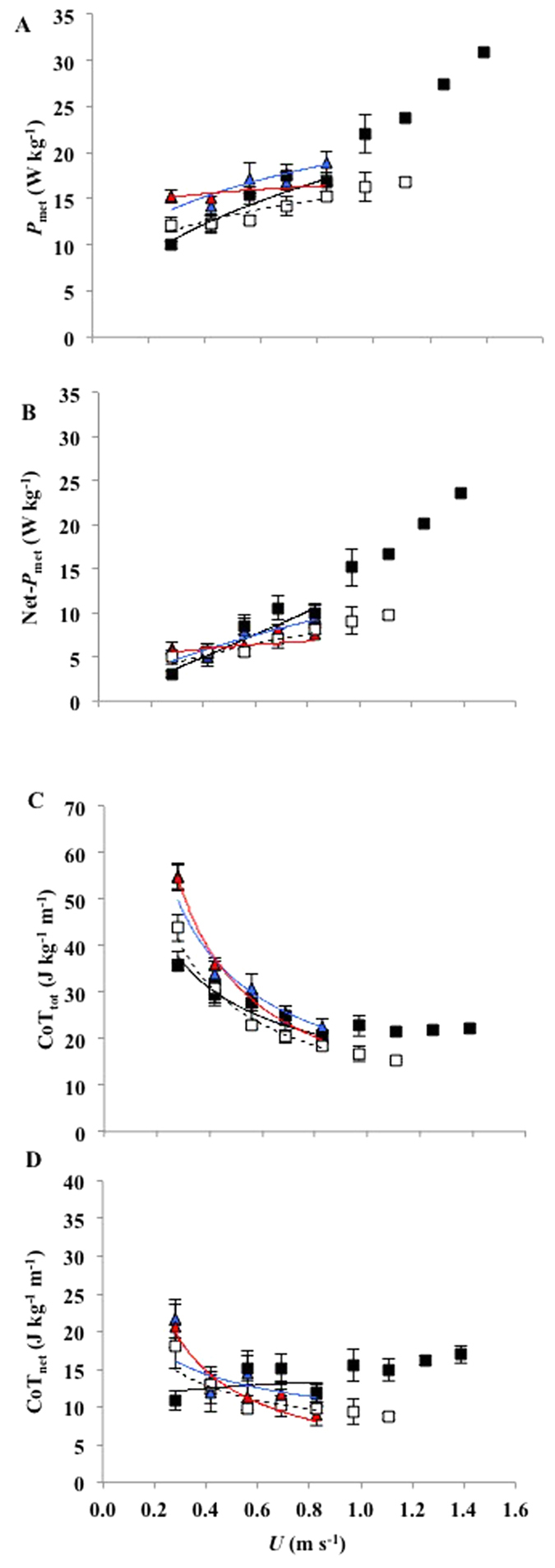
Mean mass-specific energetics parameters versus treadmill speed (*U*). **(A)** Metabolic power (*P*_met_). **(B)** Net- metabolic power (Net*−P*_met_). **(C)** Total cost of transport. **(D)** Net cost of transport. Data points are means (±s.e.m).

The incremental increase in gross mass-specific *P*_met_ (Fig. 2A) with *U* was greater in males compared to females in both cohorts (Table 4) and also greater in the mature compared to the juvenile cohort (Table 4). Following the subtracting of standing-*P*_met_ from gross locomotor *P*_met_ to calculate net-*P*_met_ (Fig. 2B), the same statistical differences were true. Therefore, faster speeds were more metabolically expensive for males than for females, and more expensive for mature, than for juvenile birds.

CoT_tot_ (Fig. 2C) decreased curvilinearly as a function of *U* in all four groups. The rate of decrease in CoT_tot_ with *U*, however, was greater in the juvenile than in the mature cohort (Table 4). The rate of decrease was also greater in females than in males. CoT_net_ (Fig. 2D) also decreased curvilinearly with *U* in all but M_♂_ in which it was invariant with speed.

### Kinematics

Each kinematic parameter responded (increased or decreased) to increases in *U* at a similar rate in all of the four bird groups, unless otherwise stated below.

**Figure 3:**
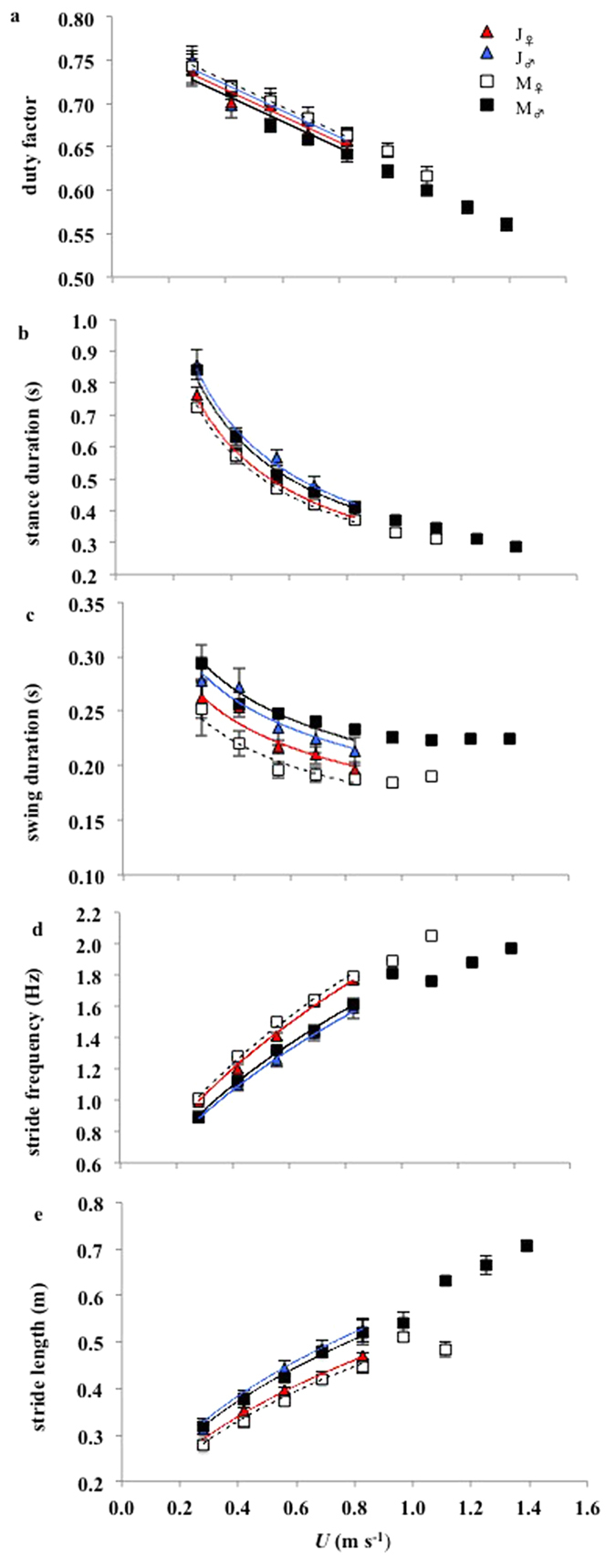
Mean kinematics parameters versus treadmill speed (*U*). **(A)** Duty factor (DF). **(B)** Stance duration (*t*_stance_). **(C)** Swing duration (*t*_swing_). **(D)** Stride frequency (*f*_stride_). **(E)** Stride length (*l*_stride_). Data points are means (±s.e.m).

Duty factor (DF, the relative contribution of the stance phase to the stride period) decreased linearly with *U.* A significant age × sex interaction was present in DF (Fig. 3A) due to DF being greater in males than in females (<1%) in the juvenile cohort but greater in females than in males in the mature cohort (by 2%) (Table 4).

Stance duration (*t*_stance_) decreased curvilinearly with *U* at a similar rate in all bird groups (Fig. 3B, Table 4). *t*_stance_ was greater in males than in females by 0.03 s in the juveniles and by 0.04 s in the mature birds; however, no significant variety × sex interaction in *t*_stance_ was identified (Table 4). Much smaller, but significant differences were also observed between the age cohorts, with *t*_stance_ being slightly greater in the juvenile compared to the mature group.

Swing duration (*t*_swing_) also decreased curvilinearly with *U* and at the same rate in all birds groups; however, *t*_swing_ (the intercept) was greater in males compared to females at any given *U* by 0.02 s in the juveniles, and by 0.04 s in the mature birds (Fig. 3C, Table 4). A significant age × sex interaction was identified in *t*_swing_ as the sex difference was greater in the mature compared to the juvenile cohort. The intercept was lower in M_♀_ relative to J_♀,_ and higher in M_♂_ relative to J_♂._ Therefore, the sexes of the mature cohort deviate in *t*_swing_ from their corresponding sexes in the juvenile cohort in different ways.

Stride frequency (*f*_stride_) increased with *U* and the trend was best described by a power function (Fig. 3D, Table 4). Sex significantly influenced *f*_stride_ (Table 4), which was 0.22 Hz and 0.23 Hz faster in females than in males across all *U* in the juvenile and mature cohorts respectively. Again, smaller but significant differences in *f*_stride_ were associated with age, which were greater in the mature compared to the juvenile cohort (by 0.06 in females and 0.05 in males).

Stride length (*l*_stride_) increased with *U* and the trend was also best described by a power function (Fig. 3E, Table 4). Strides were 0.07 m longer in males compared to females, and 0.02 m longer in the juvenile compared to the mature cohort across all *U*.

## Discussion

Here we report the first comparison of the locomotor energetics, kinematics and morphology of a species just prior to and just after the onset of sexual maturity. In comparison to a cohort of juvenile white leghorn chickens, whose sexes were similar in body form, a sexually mature cohort showed strong male-biased sexual size dimorphism, greater limb length and relative muscle mass in males and greater reproductive mass in females. Despite the large ontogenetic differences in hind limb skeletal muscle mass in males and reproductive mass in females (assumed negligible in males), no age × sex interactions in locomotor energetics were identified. The lower incremental metabolic cost of locomotion in females relative to males, common to both juvenile and sexually mature leghorns must, therefore be due to sexual dimorphisms in physiology than precede the onset of sexual maturity. An age × sex interaction was identified in only *U*_max_ and two kinematics parameters (*t*_swing_ and DF).

The sex differences in maximum performance can be linked to the measured sex differences in morphology. Maximum running speed scales with positive allometry against *M*_b_^31^; therefore the greater *U*_max_ in mature, relative to juvenile, birds and in mature males relative to females, is expected simply because of greater body size. However, the lack of sex difference in *U*_max_ in the juveniles (which were also dimorphic in leg length, but to a lesser degree) suggests a more important role of muscle in determining *U*_max_ than leg length. Muscle physiological cross sectional area (largely influenced by muscle mass) is directly proportional to the maximum force and power that a muscle can produce^32^. The greater volumes of the hind limb skeletal muscles in M_♂_ relative to the other three chicken groups, therefore, likely contributed to their greater *U*_max_ (which would require greater peak muscle forces).

*U*_max_ is not only dependent upon the maximum *f*_stride_ and *l*_stride_ that the birds can achieve, but also the ability of the birds to sustain the speed aerobically (~5min of locomotion is required for respirometry measurements). A lower *U*_max_ in juveniles is consistent with previous findings from ontogenetic comparisons, whereby stamina is usually lower in juvenile forms^24^. The greater *U*_max_ in M_♂_ may be indicative of a greater aerobic capacity. In a study on red jungle fowl, maximum rate of oxygen consumption (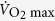) in mature males exceeded that of mature females, but no sex difference was identified in chicks^33^. Therefore, it seems likely that with the onset of sexual maturity there are physiological changes in males, which increase their capacity for aerobic respiration in the muscles. There are a number of levels at which this physiological difference could manifest. For example, in the red jungle fowl, 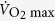 correlated with cecum, heart, pectoralis and hind limb skeletal muscle masses as well as pectoralis citrate synthase activity, indicative of system wide (peripheral and central organ) specialisation (symmorphosis)^34^.

Although there is evidence amongst vertebrate species for reductions in locomotor performance with pregnancy/gravidity^35^,^36^,^37^,^38^,^39^,^40^, *U*_max_ was actually greater in M_♀_ than in J_♀_. This is despite the two age cohorts of female sharing similar absolute limb lengths and muscle mass, whilst only M_♀_ were gravid and were also 1.36-fold heavier than J_♀_. Age differences in *U*_max_ in female leghorns are, therefore, likely explained by differences in aerobic capacity that are not linked to muscle quantity. Interestingly, in the red jungle fowl, 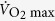 did not correlate with the mass or enzyme capacities of skeletal muscles (peripheral organs) but was correlated with haematocrit and the mass of the large intestine (a central organ)^34^.

The sex differences in most of the kinematic parameters were common to both cohorts. This is likely due to the fact that the juvenile cohort also exhibited some sexual size dimorphism in hind limb skeletal length. *t*_stance_ and *l*_stride_ were greater, and *f*_stride_ lower, in larger males relative to smaller females, as would be expected when comparing a larger animal with a smaller one (based on interspecific comparison)^41^. The sex difference in DF in the mature cohort (greater in females than in males) differed from what would be expected based on interspecific differences associated with size but can be attributed to mechano-physiological constraints imposed by the measured secondary sex characteristics. The distribution and placement of additional mass on the body have important consequences for locomotion. For example, in manipulative studies in which masses were added to the distal limbs of birds, a corresponding increase in *t*_swing_ has been reported (e.g. a 5% *M*_b_ load to the distal limb caused a ~16% increase in *t*_swing_ in the barnacle goose^42^). The greater muscle mass on the hind limbs (proximal and distal) and increase in leg length of M_♂_ relative to that of J_♂_, may increase limb inertia, which might be expected to increase *t*_swing_. Muscle mechanical work requirements are lower, the shorter the duration of the stance^43^,^44^,^45^. It is also possible that males decrease their duty factors at sexual maturity in order to minimize muscle mechanical work demands, which would be expected to increase with the rise in muscle and bone mass.

In opposition to the ontogenetic differences in *t*_swing_ found in males, *t*_swing_ was faster in M_♀_ than in J_♀_. The two groups shared a similar quantity of muscle mass but M_♀_ possessed greater reproductive mass and overall body mass to support than J_♀_. Therefore, if all kinematic parameters were identical between M_♀_ and J_♀_, peak external forces and muscle mechanical work and power might each be expected to be greater in M_♀_ compared to J_♀_. One potential reason for a faster *t*_swing_ in gravid mature females might be to increase the relative contribution of *t*_stance_ to the stride period (DF was greater in M_♀_ than all other bird groups), which would allow more time for generating sufficient muscle force to support the increase in *M*_b_. A greater DF for a given *U* would decrease peak external forces, which may be important in hens, due to a reduction in bone strength associated with the utilisation of medullary bone calcium in egg-shell formation^46^,^47^. Furthermore, a greater DF decreases muscle mechanical power requirements^43^. The ontogenetic differences in *t*_swing_ in female leghorns may, therefore, also represent a power minimizing mechanism. Loads added to the backs of birds, which increase the amount of body weight that the stance muscles must support, are not always associated with changes in kinematics parameters^42^,^48^. In a study by Marsh *et al.*^49^, however, guineafowl (*Numida meleagris*) increased their DF when carrying back loads. The location of the added load to the females during gravidity, however, has not yet been mimicked in any load carrying studies in birds. Pregnant humans^50^ and wallabies carrying young in the pouch^51^ are, like the leghorns here, known to increase DF with pregnancy.

## Conclusion

Contrary to our hypothesis that sex differences in locomotor energy metabolism would be associated with sexual maturation in white leghorns; lower incremental metabolic costs of locomotion in females relative to males, were also found in juveniles. Sexual maturation in white leghorns is associated with large increases in hind limb skeletal muscle mass in males and reproductive tissue mass in females. Differences in the location of the additional tissues on the body following sexual maturity differentially impact upon the duration of the swing phase of the limb of the sexes. We suggest that the birds modulate the swing, and hence duty factor, in order to minimize muscle mechanical work (males) and mechanical power and/or peak force (females). A role of secondary sex characteristics in influencing maximum performance in males was indicated by maximum sustainable speeds 67% greater in the mature compared to the juvenile cohort. Furthermore, no evidence was found in females for a constraint of gravidity on maximum sustainable speed. Unlike broiler chickens, which experience locomotor difficulties as they develop the muscle mass for which they were artificially selected, leghorns show a greater capacity for sustained locomotion with the onset of egg-laying.

## Methods

### Animals

Metabolic measurements from M_♂_ and M_♀_ (≥20-week-old) were taken from Rose *et al.*^30^. Juvenile (14–16 weeks-old) white leghorns (J_♂_: N = 5; *M*_b_ = 1.10 ± 0.10 kg, mean ± s.e.m) and J_♀_ (N = 7: *M*_b_ = 1.05 ± 0.03 kg, mean ± s.e.m) were obtained from the same local suppliers and housed under the same conditions in the University of Manchester’s animal unit with the same feeding regimes as the birds in Rose *et al.*^30^. None of the J_♂_ were crowing or exhibiting aggressive behaviour and J_♀_ were not gravid when examined post mortem (whilst the opposite was true for the mature cohort^30^), confirming that the birds had not reached sexual maturity. Experimental procedures were carried out under ethical approval from the University of Manchester Ethics Committee and in accordance with the Animals (Scientific Procedures) Act 1986, covered by a UK Home Office project licence (40/3549) held by Dr Codd.

### Respirometry

Metabolic rates were measured from the birds at rest (standing) and during locomotion on a motorised treadmill (Tunturi T60, Turku, Finland). An open-flow respirometry set up (described in Rose *et al.*^30^,^52^) was used to measure rates of O_2_ consumption (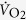, ml min^−1^) and CO_2_ production (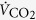, ml min^−1^). The chamber (97.5 × 53.5 × 48.0 cm) within which the birds exercised and the main flow rate (250 L min^−1^) directed through it were identical to those for the sexually mature leghorns in Rose *et al.*^30^. Juvenile respiratory exchange ratios (RERs: 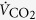: 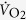) were similar to those reported for the mature cohort in Rose *et al.*^30^. Thermal equivalents^53^ of the RERs and body mass were used to convert 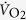 into mass-specific *P*_met_ (W kg^−1^). Metabolic rates during quiet standing were subtracted from locomotor metabolic rates to determine the net metabolic cost of locomotion surplus to maintenance and postural costs. The cost of transport (CoT_tot_, J kg^−1^ m^−1^) was calculated as *P*_met_/*U.*

The juveniles were exercised at speed intervals, up to the maximum that they could sustain: 0.28, 0.42, 0.56, 0.69 and 0.83 m s^−1^. Each bird completed two trials, composed of 2–3 speeds in a random order, interrupted by 5 min resting periods during which the birds stood quietly and gas levels plateaued. Resting (standing) metabolic rate was taken from the final rest trace in each trial.

### Kinematics

Kinematics parameters were obtained using the exact protocol used in Rose *et al.*^30^,^52^. All trials were filmed with a high-speed (100 frames s^−1^) video camera (HDR-XR520VE, Sony, Japan) from the side. The tip of digit 3 on the foot closest to the camera was tracked over ~10 strides using Tracker software (v. 4.05, Open Source Physics). Temporal data were used to calculate *f*_stride_, *l*_stride_ (*U*/*f*_stride_), *t*_swing_, *t*_stance_ and DF. Hip heights were not measured.

### Morphological measurements

Juvenile carcasses were scanned using computed tomography (CT) at the Small Animal Teaching Hospital at the University of Liverpool. Three-dimensional reconstruction of full skeletons was carried out by image segmentation and meshing in 3D Slicer (www.slicer.org). MeshLab (www.meshlab.sourceforge.net) was subsequently used to measure lengths of the femur, tibiotarsus and tarsometatarsus from the 3D skeletal models. Five frozen carcasses from each of the bird groups, excluding J_♂_ (N = 4), were defrosted for 24 hours prior to dissection. Thirteen major skeletal muscles (Table 3) were identified based on a description by Paxton *et al.*^9^ and dissected from the right pelvic limb and weighed using electronic scales (± 0.01 g).

### Statistical analyses

Only data from the range of speeds utilised by all birds (i.e. up to the *U*_max_ of the juvenile birds, 0.83 m s^−1^) were included in statistical analyses. All data from the mature birds are presented in the graphs, however, to show their capacities for *U*_max_ and associated kinematics.

All statistical analyses were performed using the car package version 2.0–12^54^ on R 2.14.0 GUI 1.42 Leopard build 64-bit^55^. Shapiro-wilk tests were performed on the standardised residuals of the models to ensure that the data approximated a normal distribution. Where the data did not conform to a normal distribution, data were log transformed. Data were also log transformed if it improved the Akaike’s information criterion of the models. Age-cohort and sex were included as fixed factors in all models. Two-way analyses of variance (ANOVAs) were used to test for differences in morphological measurements. Linear models were conducted to test for differences in the relationships between energetic or kinematic variables and *U*. *U* was included as a covariate in the models. All potential interaction terms were considered in all primary models before a step-wise backward deletion of non-significant interaction terms was conducted. The final model outputs are reported. Best-fit lines were obtained using the effect sizes from the coefficients tables output by the statistical models and were back-transformed where data had been log-transformed.

## Additional Information

**How to cite this article**: Rose, K. A. *et al.* Ontogeny of sex differences in the energetics and kinematics of terrestrial locomotion in leghorn chickens *(Gallus gallus domesticus). Sci. Rep.* **6**, 24292; doi: 10.1038/srep24292 (2016).

## Figures and Tables

**Table 1:** Mean (± s.e.m) morphological measurements.

cohort	sex	N	*M*_b_(kg)	*l*_fem_ (mm)	*l*_tib_ (mm)	*l*_tars_ (mm)	Σ*l*_segs_ (mm)
Juvenile	Female	7	1.05 ± 0.03	71.06 ± 2.03	110.01 ± 2.30	76.76 ± 1.37	257.82 ± 5.08
Juvenile	Male	5	1.10 ± 0.10	77.95 ± 2.13	120.02 ± 4.57	86.80 ± 2.46	284.78 ± 8.64
Mature	Female	7	1.43 ± 0.02	71.76 ± 1.59	108.86 ± 1.22	76.57 ± 0.87	258.10 ± 2.44
Mature	Male	5	1.92 ± 0.04	85.92 ± 1.74	129.29 ± 2.19	93.15 ± 2.36	308.37 ± 6.06

*l*_fem_, femur length; *l*_tib_, tibiotarsus length; *l*_tars_, tarsometatarsus length; Σ*l*_segs_ sum of the hind limb bone lengths.

Results of the two-way ANOVAS conducted to test for age and sex affects are in Table 2.

**Table 2:** Results of the two-way ANOVAs performed to determine whether age and sex affect the morphological measurements.

Measurement	Final model
*M*_b_	age (*F*_1,16_ = 46.53, *P* < 0.001), sex (*F*_1,16_ = 24.48, *P* < 0.001), age × sex (*F*_1,16_ = 33.83, *P* < 0.001) *R*^2^ = 0.84
*l*_fem_	age (*F*_1,21_ = 3.46, *P* = 0.077), sex (*F*_1,21_ = 26.78, *P* < 0.001) *R*^2^ = 0.55
*l*_tib_	age (*F*_1,21_ = 1.35, *P* = 0.259), sex (*F*_1,21_ = 29.69, *P* < 0.001) *R*^2^ = 0.56
*l*_tars_	age (*F*_1,21_ = 2.00, *P* = 0.172), sex (*F*_1,21_ = 53.54, *P* < 0.001) *R*^2^ = 0.70
Σ*l*_segs_	age (*F*_1,20_ = 3.36, *P* = 0.082), sex (*F*_1,20_ = 48.85, *P* < 0.001), age × sex (*F*_1,20_=4.45, *P* = 0.048) *R*^2^ = 0.70
log(*M*_IL_)	age (*F*_1,15_ = 22.56, *P* < 0.001), sex (*F*_1,15_ = 22.45, *P* < 0.001), age × sex (*F*_1,15_ = 22.87, *P* < 0.001), *R*^2^ = 0.79
*M*_IC_	age (*F*_1,15_ = 37.64, *P* < 0.001), sex (*F*_1,15_ = 26.57, *P* < 0.001), age × sex (*F*_1,15_ = 30.63, *P* < 0.001), *R*^2^ = 0.84
*M*_ILFB_	age (*F*_1,15_ = 17.23, *P* < 0.001), sex (*F*_1,15_ = 28.54, *P* < 0.001), age × sex (*F*_1,15_ = 18.35, *P* < 0.001), *R*^2^ = 0.78
*M*_FCLP_	age (*F*_1,15_ = 28.88, *P* < 0.001), sex (*F*_1,15_ = 12.85, *P* = 0.003), age × sex (*F*_1,15_ = 15.14, *P* = 0.001), *R*^2^ = 0.74
*M*_ITC_	age (*F*_1,15_ = 31.84, *P* < 0.001), sex (*F*_1,15_ = 42.15, *P* < 0.001), age × sex (*F*_1,15_ = 25.08, *P* < 0.001), *R*^2^ = 0.85
*M*_PIFL_	age (*F*_1,15_ = 19.19, *P* < 0.001), sex (*F*_1,15_ = 26.21, *P* < 0.001), age × sex (*F*_1,15_ = 17.15, *P* < 0.001), *R*^2^ = 0.78
*M*_PIFM_	age (*F*_1,15_ = 24.33, *P* < 0.001), sex (*F*_1,15_ = 41.55, *P* < 0.001), age × sex (*F*_1,15_ = 23.40, *P* < 0.001), *R*^2^ = 0.83
*M*_GL_	age (*F*_1,15_ = 46.40, *P* < 0.001), sex (*F*_1,15_ = 43.80, *P* < 0.001), age × sex (*F*_1,15_ = 47.14, *P* < 0.001), *R*^2^ = 0.89
log(*M*_GM_)	age (*F*_1,15_ = 20.49, *P* < 0.001), sex (*F*_1,15_ = 24.62, *P* < 0.001), age × sex (*F*_1,15_ = 14.84, *P* < 0.001), *R*^2^ = 0.77
*M*_FL_	age (*F*_1,15_ = 21.71 *P* < 0.001), sex (*F*_1,15_ = 25.88, *P* < 0.001), age × sex (*F*_1,15_ = 31.50, *P* < 0.001), *R*^2^ = 0.81
*M*_TCT/F_	age (*F*_1,15_ = 7.60, *P* = 0.015), sex (*F*_1,15_ = 16.84, *P* < 0.001), age × sex (*F*_1,15_ = 10.73, *P* = 0.005), *R*^2^ = 0.65
*M*_FCM_	age (*F*_1,14_ = 5.96, *P* = 0.027), sex (*F*_1,14_ = 3.40, *P* = 0.085), age × sex (*F*_1,14_ = 7.06, *P* = 0.018), *R*^2^ = 0.44
*M*_FMT_	age (*F*_1,14_ = 32.23, *P* < 0.001), sex (*F*_1,14_ = 41.18, *P* < 0.001), age × sex (*F*_1,14_ = 33.75, *P* < 0.001), *R*^2^ = 0.86
*M*_IL_: *M*_b_	age (*F*_1,15_ = 1.98, *P* = 0.180), sex (*F*_1,15_ = 8.79, *P* = 0.010), age × sex (*F*_1,15_ = 20.70, *P* < 0.001), *R*^2^ = 0.62
*M*_IC_: *M*_b_	age (*F*_1,15_ = 9.77, *P* = 0.007), sex (*F*_1,15_ = 8.04, *P* = 0.013), age × sex (*F*_1,15_ = 25.31, *P* < 0.001), *R*^2^ = 0.70
*M*_ILFB_: *M*_b_	age (*F*_1,15_ = 1.08, *P* = 0.315), sex (*F*_1,15_ = 18.52, *P* < 0.001), age × sex (*F*_1,15_ = 14.46, *P* < 0.001), *R*^2^ = 0.64
*M*_FCLP_: *M*_b_	age (*F*_1,15_ = 14.39, *P* = 0.002), sex (*F*_1,15_ = 3.67, *P* = 0.074), age × sex (*F*_1,15_ = 18.48, *P* < 0.001), *R*^2^ = 0.66
*M*_ITC_: *M*_b_	age (*F*_1,15_ = 0.03, *P* = 0.871), sex (*F*_1,15_ = 5.61, *P* = 0.032), age × sex (*F*_1,15_ = 5.21, *P* = 0.038), *R*^2^ = 0.31
*M*_PIFL_: *M*_b_	age (*F*_1,15_ = 0.13, *P* = 0.721), sex (*F*_1,15_ = 6.81, *P* = 0.020), age × sex (*F*_1,15_ = 10.16, *P* = 0.006), *R*^2^ = 0.44
*M*_PIFM_: *M*_b_	age (*F*_1,15_ = 1.58, *P* = 0.228), sex (*F*_1,15_ = 19.57, *P* < 0.001), age × sex (*F*_1,15_ = 10.87, *P* = 0.005), *R*^2^ = 0.62
*M*_GL_: *M*_b_	age (*F*_1,15_ = 5.33, *P* = 0.036), sex (*F*_1,15_ = 5.84, *P* = 0.029), age × sex (*F*_1,15_ = 25.44, *P* < 0.001), *R*^2^ = 0.66
log(*M*_GM_: *M*_b_)	age (*F*_1,15_ = 0.77, *P* = 0.395), sex (*F*_1,15_ = 12.40, *P* = 0.003), age × sex (*F*_1,15_ = 12.71, *P* = 0.003), *R*^2^ = 0.56
*M*_FL_: *M*_b_	age (*F*_1,15_ = 2.26, *P* = 0.154), sex (*F*_1,15_ = 15.48, *P* = 0.001), age × sex (*F*_1,15_ = 57.44, *P* < 0.001), *R*^2^ = 0.80
*M*_TCT/F_: *M*_b_	age (*F*_1,15_ = 0.00, *P* = 0.970), sex (*F*_1,15_ = 8.76, *P* = 0.010), age × sex (*F*_1,15_ = 6.51, *P* = 0.022), *R*^2^ = 0.41
*M*_FCM_: *M*_b_	age (*F*_1,14_ = 7.96, *P* = 0.014), sex (*F*_1,14_ = 6.62 *P* = 0.022), age × sex (*F*_1,14_ = 6.88, *P* = 0.020), *R*^2^ = 0.54
*M*_FMT_: *M*_b_	age (*F*_1,14_ = 0.79, *P* = 0.388), sex (*F*_1,14_ = 10.57 *P* = 0.006), age × sex (*F*_1,14_ = 21.88, *P* < 0.001), *R*^2^ = 0.64

The adjusted R^2^ values are reported from the final models.

**Table 3:** Pelvic limb muscles and their abbreviations and position on the limb.

Muscle	Abbreviation	Part of the limb
M. iliotibialis cranialis	IC	Proximal
M. iliotibialis lateralis (pre and post acetabularis)	IL	Proximal
M. iliofibularis	ILFB	Proximal
M. flexor cruris lateralis pars pelvica	FCLP	Proximal
M. flexor cruris medialis	FCM	Proximal
M. iliotrochantericus caudalis	ITC	Proximal
M. femerotibialis medialis	FMT	Proximal
M. pubioischiofemoralis pars lateralis	PIFL	Proximal
M. pubioischiofemoralis pars medialis	PIFM	Proximal
M. gastrocnemius pars lateralis	GL	Distal
M. gastrocnemius pars medialis	GM	Distal
M. fibularis lateralis	FL	Distal
M. tibialis cranialis caput tibiale and femorale	TCT/F	Distal

**Table 4:** Results of the final linear model outputs performed to investigate age and sex related differences in energetics and kinematics.

Parameter	Final model	Coefficients
Standing (W kg^−1^)	age (*F*_1,23_ = 13.86, *P* = 0.001),	J_F_: = 9.44
	sex (*F*_1,23_ = 0.16, *P* = 0.691)	J_M_: = 9.19
	*R*^2^ = 0.33	M_F_: = 7.17
		M_M_: = 6.92
log *P*_met_ (W kg^−1^)	log*U* (*F*_1,108_ = 53.25, *P* < 0.001),	J_F_: = 16.62*U*^0.07^
	age (*F*_1,108_ = 40.55, *P* < 0.001),	J_M_: = 19.70*U*^0.28^
	sex (*F*_1,108_ = 1.39, *P* = 0.241),	M_F_: = 15.75*U*^0.25^
	log*U* × age (*F*_1,108_ = 6.25, *P* = 0.014),	M_M_: = 18.67*U*^0.46^
	log*U* × sex *F*_1,108_ = 8.53, *P* = 0.004),	
	*R*^2^ = 0.48	
log Net-*P*_met_ (W kg^−1^)	log*U* (*F*_1,108_ = 50.93, *P* < 0.001),	J_F_: = 7.08*U*^0.20^
	age (*F*_1,108_ = 0.57, *P* = 0.451),	J_M_: = 10.46*U*^0.66^
	sex (*F*_1,108_ = 1.71, *P* = 0.194),	M_F_: = 8.63*U*^0.57^
	log*U* × age (*F*_1,108_ = 4.91, *P* = 0.029),	M_M_: = 12.74*U*^1.03^
	log*U* × sex (*F*_1,108_ = 7.49, *P* = 0.007)	
	*R*^2^ = 0.35	
log CoT_tot_ (J kg^−1^ m^−1^)	log*U* (*F*_1,108_ = 462.05, *P* < 0.001),	J_F_: = 16.51*U*^−0.94^
	age (*F*_1,108_ = 40.71, *P* < 0.001),	J_M_: = 19.58*U*^−0.73^
	sex (*F*_1,108_ = 1.41, *P* = 0.237),	M_F_: = 18.56*U*^−0.76^
	log*U* × age (*F*_1,108_ = 6.32, *P* = 0.013),	M_M_: = 15.65*U*^−0.55^
	log*U* × sex (*F*_1,108_ = 8.60, *P* < 0.001),	
	*R*^2^ = 0.82	
log CoT_net_ (J kg^−1^ m^−1^)	log*U* (*F*_1,108_ = 25.39, *P* < 0.001),	J_F_: = 6.93*U*^−0.83^
	age (*F*_1,108_ = 0.57, *P* = 0.454),	J_M_: = 10.60*U*^−0.33^
	sex (*F*_1,108_ = 1.72, *P* = 0.193),	M_F_: = 8.82*U*^−0.41^
	log*U* × age (*F*_1,108_ = 4.93, *P* = 0.028),	M_M_: = 13.49*U*^−0.09^
	log*U* × sex (*F*_1,108_ = 7.50, *P* = 0.007)	
	*R*^2^ = 0.24	
DF	*U* (*F*_1,100_ = 138.38, *P* < 0.001),	J_F_: = −0.15*U*+0.78
	age (*F*_1,100_ = 0.02, *P* = 0.898),	J_M_: = −0.15*U*+0.78
	sex (*F*_1,100_ = 1.08, *P* = 0.302),	M_F_: = 0.15*U*+0.79
	age × sex (*F*_1,100_ = 5.02, *P* = 0.027)	M_M_: = 0.15*U*+0.77
	*R*^2^ = 0.58	
log *t*_stance_ (s)	log*U* (*F*_1,101_ = 1322.38, *P* < 0.001),	J_F_: = 0.34*U*^−0.64^
	age (*F*_1,101_ = 5.87, *P* = 0.017),	J_M_: = 0.37*U*^−0.64^
	sex (*F*_1,101_ = 62.84, *P* < 0.001)	M_F_: = 0.32*U*^−0.64^
	*R*^2^ = 0.93	M_M_: = 0.36*U*^−0.64^
log *t*_swing_ (s)	log*U* (*F*_1,100_ = 98.18, *P* < 0.001),	J_F_: = 0.19*U*^−0.26^
	age (*F*_1,100_ = 2.37, *P* = 0.127),	J_M_: = 0.21*U*^−0.26^
	sex (*F*_1,100_ = 43.75, *P* < 0.001),	M_F_: = 0.18*U*^−0.26^
	age × sex (*F*_1,100_ = 8.05, *P* < 0.006)	M_M_: = 0.22*U*^−0.26^
	*R*^2^ = 0.59	
log *f*_stride_ (Hz)	log*U* (*F*_1,101_ = 1252.22, *P* < 0.001),	J_F_: = 1.95*U*^0.53^
	age (*F*_1,101_ = 5.23, *P* = 0.024),	J_M_: = 1.73*U*^0.53^
	sex (*F*_1,101_ = 103.25, *P* < 0.001)	M_F_: = 2.01*U*^0.53^
	*R*^2^ = 0.93	M_M_: = 1.78*U*^0.53^
log *l*_stride_ (m)	log*U* (*F*_1,101_ = 849.43, *P* < 0.001),	J_F_: = 0.51*U*^0.44^
	age (*F*_1,101_ = 6.02, *P* = 0.016),	J_M_: = 0.58*U*^0.44^
	sex (*F*_1,101_ = 98.97, *P* < 0.001)	M_F_: = 0.49*U*^0.44^
	*R*^2^ = 0.93	M_M_: = 0.56*U*^0.44^

The adjusted R^2^ values are reported from the final models.
